# WNT/β-catenin-suppressed FTO expression increases m^6^A of c-Myc mRNA to promote tumor cell glycolysis and tumorigenesis

**DOI:** 10.1038/s41419-021-03739-z

**Published:** 2021-05-08

**Authors:** Xueying Yang, Fei Shao, Dong Guo, Wei Wang, Juhong Wang, Rongxuan Zhu, Yibo Gao, Jie He, Zhimin Lu

**Affiliations:** 1 Department of Thoracic Surgery, National Cancer Center/National Clinical Research Center for Cancer/Cancer Hospital, Chinese Academy of Medical Sciences and Peking Union Medical College, 100021 Beijing, China; 2 The Affiliated Hospital of Qingdao University and Qingdao Cancer Institute, 266071 Qingdao, Shandong China; 3 Zhejiang Provincial Key Laboratory of Pancreatic Disease, The First Affiliated Hospital, and Institute of Translational Medicine, Zhejiang University School of Medicine, 310029 Hangzhou, China; 4 State Key Laboratory of Molecular Oncology, National Cancer Center/National Clinical Research Center for Cancer/Cancer Hospital, Chinese Academy of Medical Sciences and Peking Union Medical College, 100021 Beijing, China; 5 Zhejiang University Cancer Center, 310029 Hangzhou, China

**Keywords:** Non-small-cell lung cancer, Epigenetics

## Abstract

FTO removes the N6-methyladenosine (m^6^A) modification from genes and plays a critical role in cancer development. However, the mechanisms underlying the regulation of FTO and its subsequent impact on the regulation of the epitranscriptome remain to be further elucidated. Here, we demonstrate that FTO expression is downregulated and inversely correlated with poor survival of lung adenocarcinoma patients. Mechanistically, Wnt signaling induces the binding of EZH2 to β-catenin. This protein complex binds to the LEF/TCF-binding elements at the promoter region of *FTO*, where EZH2 enhances H3K27me3 and inhibits FTO expression. Downregulated FTO expression substantially enhances the m^6^A levels in the mRNAs of a large number of genes in critical pathways, particularly metabolic pathway genes, such as *MYC*. Enhanced m^6^A levels on *MYC* mRNA recruit YTHDF1 binding, which promotes *MYC* mRNA translation and a subsequent increase in glycolysis and proliferation of tumor cells and tumorigenesis. Our findings uncovered a critical mechanism of epitranscriptome regulation by Wnt/β-catenin-mediated FTO downregulation and underscored the role of m^6^A modifications of *MYC* mRNA in regulating tumor cell glycolysis and growth.

## Introduction

N6-Methyladenosine (m^6^A) is the most abundant and reversible posttranscriptional modification of mRNAs in eukaryotes^1,2^. m^6^A modifications have an important role in RNA splicing, stability, transport, and translation^3–7^. The abundance and effects of m^6^A on RNA depend on the dynamic and integrated regulation by “writers” and “erasers”, which add and remove the methylation, respectively, and “readers”, which recognize the modification^2,8^. The identified writers include methyltransferase-like (METTL) 3/14, Wilms tumor 1-associated protein (WTAP), RNA binding motif protein 15/15B (RBM15/15B), and KIAA1429, whereas erasers include fat mass and obesity-associated protein (FTO) and alkB homolog 5 (ALKBH5). YT521-B homology (YTH) domain-containing proteins (YTHDF1-3, YTHDC1-2), heterogeneous nuclear ribonucleoprotein (HNRNP) protein families, and IGF2 mRNA binding proteins (IGF2BP) families are regarded as readers^8,9^. FTO, as the first m^6^A demethylase identified^10,11^, regulates the m^6^A modification of critical genes in different types of cancer, such as glioblastoma^12^, acute myeloid leukemia (AML)^13,14^, cervical squamous cell carcinoma (CSCC)^15^, and breast cancer^16^. However, the mechanisms underlying the regulation of FTO and its subsequent impact on the regulation of the epitranscriptome remain to be further elucidated.

In this study, we demonstrate that Wnt signaling induces complex formation between the histone methyltransferases EZH2 and β-catenin, which bind to the promoter region of *FTO*, increase H3K27me3 levels, and inhibit FTO expression. Downregulated FTO expression substantially increases the m^6^A modifications on *MYC*, resulting in the binding of YTHDF1 to promote *MYC* mRNA translation and tumor cell glycolysis and growth.

## Materials and methods

### Materials

The following antibodies were purchased from Cell Signaling Technology (Danvers, MA): normal rabbit IgG (# 2729) (for immunoprecipitation and ChIP), β-catenin (#8480) (for immunoblotting and immunoprecipitation), FTO (#31687) (for immunoblotting and RIP), EZH2 (#5246) (for immunoprecipitation and ChIP), TCF4 (#2569) (for ChIP), H3K27me3 (#9733) (for ChIP), H3K9me2 (#4658) (for ChIP), HK2 (#2867) (for immunoblotting and IHC), and Ki-67 (#9449) (for IHC). A mouse monoclonal antibody against tubulin (T9026) (for immunoblotting) was purchased from Sigma-Aldrich (St. Louis, MO). Rabbit antibodies recognizing c-Myc (ab32072) (for immunoblotting and IHC), Wnt-3a (ab219412) (for IHC) and FTO (ab124892) (for IHC) were purchased from Abcam (Cambridge, MA). Rabbit antibody recognizing YTHDF1 (17479-1-AP) (for RIP and immunoblotting) was purchased from Proteintech (IL, USA). RIPA lysis and extraction buffer (89901) and Pierce IP lysis buffer (87787) were purchased from Thermo Fisher Scientific (Waltham, MA). Protein A/G plus-agarose (sc-2003) was purchased from Santa Cruz Biotechnology (Santa Cruz, CA). Wnt-3a (5036-WN) was obtained from R&D Systems (Minneapolis, MN). Cycloheximide (CHX) (HY-12320) and nitro blue tetrazolium chloride (HY-15925) were purchased from MedChem Express (Monmouth Junction, NJ). Agar (1182GR500) was purchased from BIO FROXX (Guangzhou, China).

### Data resource

The clinical records and RNAseqV2 level 3 gene-level lung adenocarcinoma data were downloaded from TCGA (http://xena.ucsc.edu/welcome-to-ucsc-xena/). Gene transcription estimates for each gene were analyzed with RNA-Seq using Expectation Maximization (RSEM) software.

### Specimens and cell lines

Forty pairs of frozen tissues for RNA extraction and 83 pairs of frozen tissues for immunohistochemistry (IHC) were obtained from patients with lung adenocarcinoma who underwent radical resections in the Department of Thoracic Surgery of the Cancer Hospital, Chinese Academy of Medical Sciences. The clinical features of the patients are summarized in Table S1. We acquired completed follow-up information for 83 patients. The time from the date of diagnosis to death or the last known date of follow-up was defined as overall survival (OS). All paired tumor and adjacent normal tissues used in this study were collected with informed consent. This study was approved by the Ethics Committee of the National Cancer Center/Cancer Hospital, Chinese Academy of Medical Sciences, and Peking Union Medical College. HEK 293T and H322 and H358 lung adenocarcinoma cells were obtained from ATCC.

### Tissue microarray and immunohistochemistry analysis

Eighty-three pairs of frozen tissues from lung adenocarcinoma patients were formalin-fixed and paraffin-embedded. The tissue microarray (TMA) was constructed as previously described^17^. Section of lung adenocarcinoma TMA was stained with an antibody against FTO. The tissue sections were quantitatively scored according to the percentage of positive cells and staining intensity as described previously^18^. The following proportion scores were assigned to the sections: 1, 0–1%; 2, 2–10%; 3, 11–30%; 4, 31–70%; and 5, 71–100%. The staining intensity was rated on a scale of 0–3: 0, negative; 1, weak; 2, moderate, and 3, strong. Then the proportion and intensity scores were combined to obtain a total score (range, 0–8) as described previously^18^.

### Cell culture

H322 and HEK 293T cells were grown in Dulbecco’s modified Eagle’s medium (DMEM) supplemented with 10% fetal bovine serum (Invitrogen) and 1% penicillin-streptomycin. H358 cells were grown in RPMI 1640 supplemented with 10% FBS (Invitrogen) and 1% penicillin-streptomycin. Cells were cultured in 5% CO_2_ at 37 °C in a humidified incubator. And all these cells were routinely tested for mycoplasma. When cells were 50% confluent, the medium was replaced with a fresh medium containing 0.5% serum for 1 day, and then Wnt-3a was added at a final concentration of 60 ng/ml for cell stimulation.

### RNA extraction and quantitative RT-PCR analysis

Total RNA was isolated with TRIzol reagent (Invitrogen, USA) according to the manufacturer’s instructions. RNA (1000 ng) was reverse-transcribed into cDNA with a RevertAid First Strand cDNA Synthesis kit (Thermo); SYBR Green-based qRT-PCR was performed using a 7900HT fast real-time PCR system (Applied Biosystems/Life Technologies, Waltham, USA), as described previously^19^. The relative mRNA expression levels were calculated by the 2^−ΔΔCt^ method with normalization to ACTB or GAPDH; the PCR primers are listed in Table S2.

### Lentivirus production and infection

Plasmids containing transgenes and packaging plasmids were cotransfected into HEK 293T cells using Lipofectamine 3000 (Invitrogen, USA). Viruses were collected and concentrated after 48 h. When tumor cells reached 50%-60% confluence, we infected the cells with concentrated virus and then selected them by antibiotic treatment^20^. The shRNA sequences are listed in Table S3.

### Immunoblotting and immunoprecipitation analysis

Extraction of proteins with a modified buffer from cultured cells was followed by immunoprecipitation and immunoblotting with antibodies, as described previously^21^.

### Chromatin immunoprecipitation (ChIP) assays

ChIP assays were performed using the SimpleChIP® Enzymatic Chromatin IP kit (#9003, Cell Signaling Technology, Danvers, USA) according to the manufacturer’s instructions, as described previously^22^. The primers are listed as follows:

binding site within the *FTO* promoter Forward: GTTATCCTTCTTTGCTCACTATGC;

binding site within the *FTO* promoter Reverse: CTGAGGAAGTGAACTGAGCTC.

### Dual-luciferase reporter assays

For the promoter-reporter assay, the wild-type DNA oligos of the FTO promoter and the mutated oligos with three LEF/TCF-binding element (TBE) deletions were inserted into the upstream region of the firefly luciferase of the PGL4.1 vector. For m^6^A reporter assays, the DNA fragments of MYC-CDS containing the wild-type m^6^A motifs, as well as the mutated motifs (m^6^A was replaced by T) were inserted into the Xhol site of the pMIR-REPORT luciferase reporter vector. Dual-luciferase reporter assays were performed, as described previously in HEK 293T cells^23^.

Sequences of wild type FTO promoter:

GGAAGTACTCCTATAGAAAAGGTCAATTTTTAGGATCCTGTTGACACATA GGCCCGTGTATGAAAATGATTAGTTTTCCATGACAGAGTTAAGGTCACTT TAAAAATAATAATGATGATGATGATGATGATGGTGTTAACATTCATTGAA CGCTTACTATGTGCCAGGTACTGTTCTAAGTGTTCTGTTATAGGAATGAA GTGTCTCACCATATCCTTGTGAGGCTGTTACTCAAATGATTCCTGCTTTA CAAATGAGGAAGCTGAGACACAGATTAGTTAACTCACTTAAGGTGGTAGT TGAAAGTATTAATAGTTGTGTCTGGTGATATTTTTGGTTGTCACAACAAG GAAGCGGGATGCTACTGGCATCTAGTGAGTAGAGGCCATGGATGATGCTA AATATCTTACAGTGCTTAGGAGACATAATAATGAATTATCCAGCCCAAAA TGTTAATAATAGTGTAGAAGCTGAAAAACCCTGCACAATGCTGCAATGCC TCTCCAACACCATCTTATGTTATCCTT**CTTTGCTC**ACTATGCTTCACTTA CATTATTCTTTACTTTCCTCGAACCCCCCATACCCTTGTCTTGCTCAAGG C**CTTTGTAT**TAGCTGGTTCCTTAAT**CTTTGGAG**CTCAGTTCACTTCCTCA GACAGGTTTTCCCTGACCATCCTATGTTAGAGTAGTCTTCCTTACATTTC TTCACTGTTTATTTCTTTTCTTTTCTTTTTTTTTTTTTTGAGACAGGGTC TTGCTATGTTGCCCGGGCTGGCCTTGAATTCATGGGTTCAAGTGATCCTC CCACCTCAGCCTCCCGAGTAGCTGGAACTACATGTGCGTGCCACCAAGCA TGGCTTGTATCTCTTATAGCAACTGCCTCTATCTGAAGTTATCAGATAAA ATTATTGTTTGTCTCCACTAAAAAAGGATAAACATCTTGAGACGGGTATT AGTCTTGTTCACAACTGTTCAGGAACAGTGCCTGGTACAGGGTGGGAACC AACATTAATATTTATTGAATGATTGGCTGTGCGTGGTGGCTCACACCTGT AATCCCAGCACTTTGCGAGGCCGAGACGGGCGGCTGACTTCAGGCCAGGA GTTCGAGACCAGCCTGGCCAACATGATGAAACCCTGTCTCTACTAAAAAT ACAAAAATTAGCTGGGTGTGGTGGCACACGCCTGTAATCCCAGCTACTCG GGAGGCTGAGGCAGAAGAATCGCTTGAACCTGGGAGGAGGAGTTTGCAGT AAGCTGAGGTCTTACCACTGCACTCCAGCCTGGGCAACGGAGCAAGAACC TGTCACACACACAAAAAAAAGAATAAAGAAAAAATATTTATTGAATGAAT AAATGAATATCAGGTACTGAGATTAAAATGGCAAGCAAAACCCCCGCCTT TATGAAGCTAGCAAGTTATGGAGGTAATCACATGATAAACAAATAATATA TAATTAAGCAAACAATAGACCACTCAGGAGGTTTAGGGTCTACCAACCAA CTCCTAATCCAGGGCAAATGAGCAAACTGTGTTAGGGACCTACAACTTGC AGGATCTGGATAGAGATGGCAATTAGCAGCATCAACTCTCACCTTCATGG CTGGGATATAACATTTCAAATTGGTCCTGGACGTGGGGATAAAGGGCGGC CTGTGATTCAGGCCTGAGGATGTGGAGGTGTCTTGGGCTGGGCTGCTTTC ACGCCAGCAGAACTCCAGGGCCAACTCCAGGGCCTTCTCCAGGCGGCAGA GCGGACCCTAGGACCCCGGCCCGCGCTGCAGTGGGGAGGGTCAGCAACCT CCACCCACCCTCATCCTCCCCCATCCTCCCGGGTACTCACCGTGCCACTG GCCCTGCAGCTAGCTACCGTTGCTATAGCGCCGACAGCGTGGCGGGCGGC TGGCCGAGAGGAGCACGGGAGAAACATGGCAGGCTCCCGTAGCCTCCTGG GAAATGTAGTTCTCCTTGGACTCTAGCCTGTTTGCTCGCGGGGTAGCGGA CTCATTTATGCTTGGTGTTATGATTGTAACTAAGAATCCTGGAGTGAGCT GGTTACAAAGTGAGCCCGACTTTCCATGGATGCACCATCCTAGAGTGCAC

Sequences of truncated FTO promoter:

GGAAGTACTCCTATAGAAAAGGTCAATTTTTAGGATCCTGTTGACACATA GGCCCGTGTATGAAAATGATTAGTTTTCCATGACAGAGTTAAGGTCACTT TAAAAATAATAATGATGATGATGATGATGATGGTGTTAACATTCATTGAA CGCTTACTATGTGCCAGGTACTGTTCTAAGTGTTCTGTTATAGGAATGAA GTGTCTCACCATATCCTTGTGAGGCTGTTACTCAAATGATTCCTGCTTTA CAAATGAGGAAGCTGAGACACAGATTAGTTAACTCACTTAAGGTGGTAGT TGAAAGTATTAATAGTTGTGTCTGGTGATATTTTTGGTTGTCACAACAAG GAAGCGGGATGCTACTGGCATCTAGTGAGTAGAGGCCATGGATGATGCTA AATATCTTACAGTGCTTAGGAGACATAATAATGAATTATCCAGCCCAAAA TGTTAATAATAGTGTAGAAGCTGAAAAACCCTGCACAATGCTGCAATGCC TCTCCAACACCATCTTATGTTATCCTTACTATGCTTCACTTA CATTATTCTTTACTTTCCTCGAACCCCCCATACCCTTGTCTTGCTCAAGG CTAGCTGGTTCCTTAATCTCAGTTCACTTCCTCA GACAGGTTTTCCCTGACCATCCTATGTTAGAGTAGTCTTCCTTACATTTC TTCACTGTTTATTTCTTTTCTTTTCTTTTTTTTTTTTTTGAGACAGGGTC TTGCTATGTTGCCCGGGCTGGCCTTGAATTCATGGGTTCAAGTGATCCTC CCACCTCAGCCTCCCGAGTAGCTGGAACTACATGTGCGTGCCACCAAGCA TGGCTTGTATCTCTTATAGCAACTGCCTCTATCTGAAGTTATCAGATAAA ATTATTGTTTGTCTCCACTAAAAAAGGATAAACATCTTGAGACGGGTATT AGTCTTGTTCACAACTGTTCAGGAACAGTGCCTGGTACAGGGTGGGAACC AACATTAATATTTATTGAATGATTGGCTGTGCGTGGTGGCTCACACCTGT AATCCCAGCACTTTGCGAGGCCGAGACGGGCGGCTGACTTCAGGCCAGGA GTTCGAGACCAGCCTGGCCAACATGATGAAACCCTGTCTCTACTAAAAAT ACAAAAATTAGCTGGGTGTGGTGGCACACGCCTGTAATCCCAGCTACTCG GGAGGCTGAGGCAGAAGAATCGCTTGAACCTGGGAGGAGGAGTTTGCAGT AAGCTGAGGTCTTACCACTGCACTCCAGCCTGGGCAACGGAGCAAGAACC TGTCACACACACAAAAAAAAGAATAAAGAAAAAATATTTATTGAATGAAT AAATGAATATCAGGTACTGAGATTAAAATGGCAAGCAAAACCCCCGCCTT TATGAAGCTAGCAAGTTATGGAGGTAATCACATGATAAACAAATAATATA TAATTAAGCAAACAATAGACCACTCAGGAGGTTTAGGGTCTACCAACCAA CTCCTAATCCAGGGCAAATGAGCAAACTGTGTTAGGGACCTACAACTTGC AGGATCTGGATAGAGATGGCAATTAGCAGCATCAACTCTCACCTTCATGG CTGGGATATAACATTTCAAATTGGTCCTGGACGTGGGGATAAAGGGCGGC CTGTGATTCAGGCCTGAGGATGTGGAGGTGTCTTGGGCTGGGCTGCTTTC ACGCCAGCAGAACTCCAGGGCCAACTCCAGGGCCTTCTCCAGGCGGCAGA GCGGACCCTAGGACCCCGGCCCGCGCTGCAGTGGGGAGGGTCAGCAACCT CCACCCACCCTCATCCTCCCCCATCCTCCCGGGTACTCACCGTGCCACTG GCCCTGCAGCTAGCTACCGTTGCTATAGCGCCGACAGCGTGGCGGGCGGC TGGCCGAGAGGAGCACGGGAGAAACATGGCAGGCTCCCGTAGCCTCCTGG GAAATGTAGTTCTCCTTGGACTCTAGCCTGTTTGCTCGCGGGGTAGCGGA CTCATTTATGCTTGGTGTTATGATTGTAACTAAGAATCCTGGAGTGAGCT GGTTACAAAGTGAGCCCGACTTTCCATGGATGCACCATCCTAGAGTGCAC

MYC-CDS with wild-type m^6^A sites:

GTAGTTATCCTTAAAAAAGCCACAGCATACATCCTGTCCGTCCAAGCAGAGGAGCAAAAGCTCATTTCTGAAGA**GGACT**TGTTGCGGAAACGACGA**GAACA**GTTG**AAACA**C**AAACT**T**GAACA**GCTACG**GAACT**CTTGTGCG

MYC-CDS with the mutated m^6^A sites:

GTAGTTATCCTTAAAAAAGCCACAGCATACATCCTGTCCGTCCAAGCAGAGGAGCAAAAGCTCATTTCTGAAGA**GGTCT**TGTTGCGGAAACGACGA**GATCA**GTTG**AATCA**C**AATCT**T**GATCA**GCTACG**GATCT**CTTGTGCG.

### Cell proliferation assay

2 × 10^5^ cells were seeded in a 6-well plate and maintained in a medium with 10% FBS for different periods of time. The cells were harvested and counted^24^.

### Soft agar assay

The assay was performed as described previously^25^. Briefly, for the bottom layer of agar, we deposited the mix of 1% agar and 2× medium into each well of a six-well plate. For the upper layer of agar, we deposited the mix of 0.6% agar and a suspension of cells in each well (10000 cells/well). The cells were cultured for 21 days in a 37 °C humidified cell culture incubator and then were stained with nitroblue tetrazolium chloride solution (200 μl/ well) and incubated overnight at 37 °C.

### Migration and invasion assays

Migration and invasion assays were performed in the chamber coated with or without Matrigel matrix (24 well, 8 μm pore size, Corning, USA) according to the manufacturer’s instructions as previously described^26^.

### Cell synchronization and cell cycle detection

2 × 10^6^ Cells were seeded in 6 cm plates. After incubation for 24 h, the culture medium was replaced and the cells were arrested at the S phase by adding thymidine (2 mM) for 24 h. The thymidine was then removed, and the cells were washed by PBS and cultured in a fresh culture medium for 3 h to release cells before nocodazole treatment (100 ng/ml) for 12 h to arrest cells in the G2/M phase. Then, the cells were washed with PBS and cultured in a fresh medium to release cells^27^.

One hour before harvesting cells at each time point, BrdU (ThermoFisher, B23151) (20 μM) was used to label the cells. The cells were collected by centrifugation at 500 × *g* for 5 min, followed by washing with PBS. The cells were then fixed in 1 ml ice-cold 70% ethanol at 4 °C for 24 h and centrifuged at 500 × *g* for 5 min. After being washed with PBS, the fixed cells were fully resuspended in 1 ml of 2 M HCl/0.5% Triton X-100 solution and incubated at room temperature for 30 min. The cells were spun down and neutralized in 1 ml of 0.1 M Na2B4O7 solution (pH, 8.5). Then, the cells were spun down and washed with cold PBS containing 1% BSA and incubated with mouse anti-BrdU (BD PharMingen, Cat#347580, 1:300) at 4 °C for 12 h. Afterward, centrifuged cells were washed with cold PBS containing 1% BSA and incubated with Alexa Fluor 647-conjugated goat anti-mouse IgG (Invitrogen, Cat#A21235, 1:400) in room temperature for 1 h. Finally, the cells were spun down and washed with cold PBS containing 1% BSA, followed by resuspended in 300 μl of PBS containing 5 μg/ml DAPI for 15 min at room temperature, and then cell cycle progression was analyzed by flow cytometry.

### Measurements of glucose consumption and lactate production

Cells were seeded in culture dishes, and the medium was changed when cells reached 50% confluence. After incubation for 12–24 h, the culture medium was collected. The glucose levels were detected by a glucose colorimetric assay kit (#K606, BioVision), and the lactate levels were detected by a lactate colorimetric assay kit (#K627, BioVision) according to the manufacturer’s instructions, and values were calculated as previously described^18^.

### Animal experiments

Mice were randomized into several groups. For the subcutaneous implantation model, 1 × 10^6^ cells were injected subcutaneously into the flank regions of female BALB/c nude mice (4–5 weeks). The width (W) and length (L) of the tumors were measured every three days, and the volume (V) of each tumor was calculated using the formula V = (W^2 ^× L/2). For lung colonization assays, 1 × 10^6^ cells were injected into the tail vein of female NOD/SCID mice (6–7 weeks), and 6 weeks later the lung was removed and fixed with 10% formalin. Fixed lung tissues were embedded in paraffin and cut into consecutive sections. These sections were stained by hematoxylin and eosin (H&E)^28^. All animal experiments were approved by the Animal Care and Use Committee of the Cancer Hospital of the Chinese Academy of Medical Sciences.

### RNA immunoprecipitation (RIP) assays

A Magna RIP kit (17–700, Millipore, MA) was used to perform the RIP assays. A sufficient number of H322 cells were lysed by RIP lysis buffer, and the supernatant of the RIP lysate was incubated with specific antibodies on beads overnight at 4 °C. After washing, RNA was extracted and analyzed by qRT-PCR.

### m^6^A-seq assays and data analysis

m^6^A-seq was performed by Cloudseq Biotech Inc. (Shanghai, China) according to the published procedure with slight modifications^1^. Briefly, fragmented mRNA was incubated with an anti-m^6^A polyclonal antibody (Synaptic Systems, 202003) in IPP buffer for 2 h at 4 °C. The mixture was then immunoprecipitated by incubation with protein-A beads (Thermo Fisher) at 4 °C for an additional 2 h. The bound RNA was eluted from the beads with N6-methyladenosine (Berry and Associates, PR3732) in IPP buffer, and then the RNA was extracted with TRIzol reagent (Thermo Fisher) following the manufacturer’s instructions. Purified RNA was used for RNA-seq library generation with the NEBNext® Ultra™ RNA Library Prep kit (NEB). Both the input sample without immunoprecipitation and the m^6^A IP samples were subjected to 150 bp paired-end sequencing on an Illumina HiSeq 4000 sequencer.

Paired-end reads were harvested from Illumina HiSeq 4000 sequencer and were quality-controlled by Q30. After 3′ adapter-trimming and low-quality reads removal by cutadapt software (v1.9.3), clean reads of input libraries were aligned to reference genome (UCSC HG19) by STAR software^29^. After that, clean reads of all libraries were aligned to the reference genome by Hisat2 software (v2.0.4)^30^. Methylated sites on RNAs (peaks) were identified by MACS software^31^. Differentially methylated sites were identified by diffReps^32^. These peaks identified by both software and overlapped with exons of mRNAs were selected. Raw counts of mRNA sequencing were got by HTSeq software (v0.9.1) and normalized by edgeR software.

### m^6^A qPCR

Total RNA was isolated with a miRNeasy kit (#217004, QIAGEN) with DNase I digestion. mRNA was extracted from total RNA using a GenElute mRNA Miniprep kit (MRN10, Sigma-Aldrich). Then, a Magna MeRIP m^6^A kit (#17–10499, Millipore) was used according to the manufacturer’s instructions. m^6^A enrichment was analyzed by qPCR with specific primers, and data were normalized to input. Primer sequences were as follows:

MYC Forward: TTGCGGAAACGACGAGAACA;

MYC Reverse: TCATAGGTGATTGCTCAGGACA.

### Polysome profiling

Cycloheximide (CHX) was added to the cell culture at a final concentration of 100 μg/ml for 10 min to stop translation. Cells were washed with cold PBS and lysed with lysis buffer. The cell lysate was loaded onto the top of a 10–50% sucrose gradient tube immediately, and the tube was centrifuged at 36,000 rpm for 3.5 h at 4 °C. The sample was separated into 12 fractions by a fraction collector and measured at 254 nm. RNA was extracted by TRIzol and subjected to qPCR analysis. The relative expression of MYC in each fraction was normalized to GAPDH, as well as to the input^33^.

### Statistics

All data are expressed as the mean ± SD. We used two-tailed Student’s *t*-tests to compare means between two groups; *p* < 0.05 was considered significant.

## Results

### FTO expression is downregulated and inversely correlated with lung adenocarcinoma patient poor survival and promotes tumor growth and metastasis

**Fig. 1:**
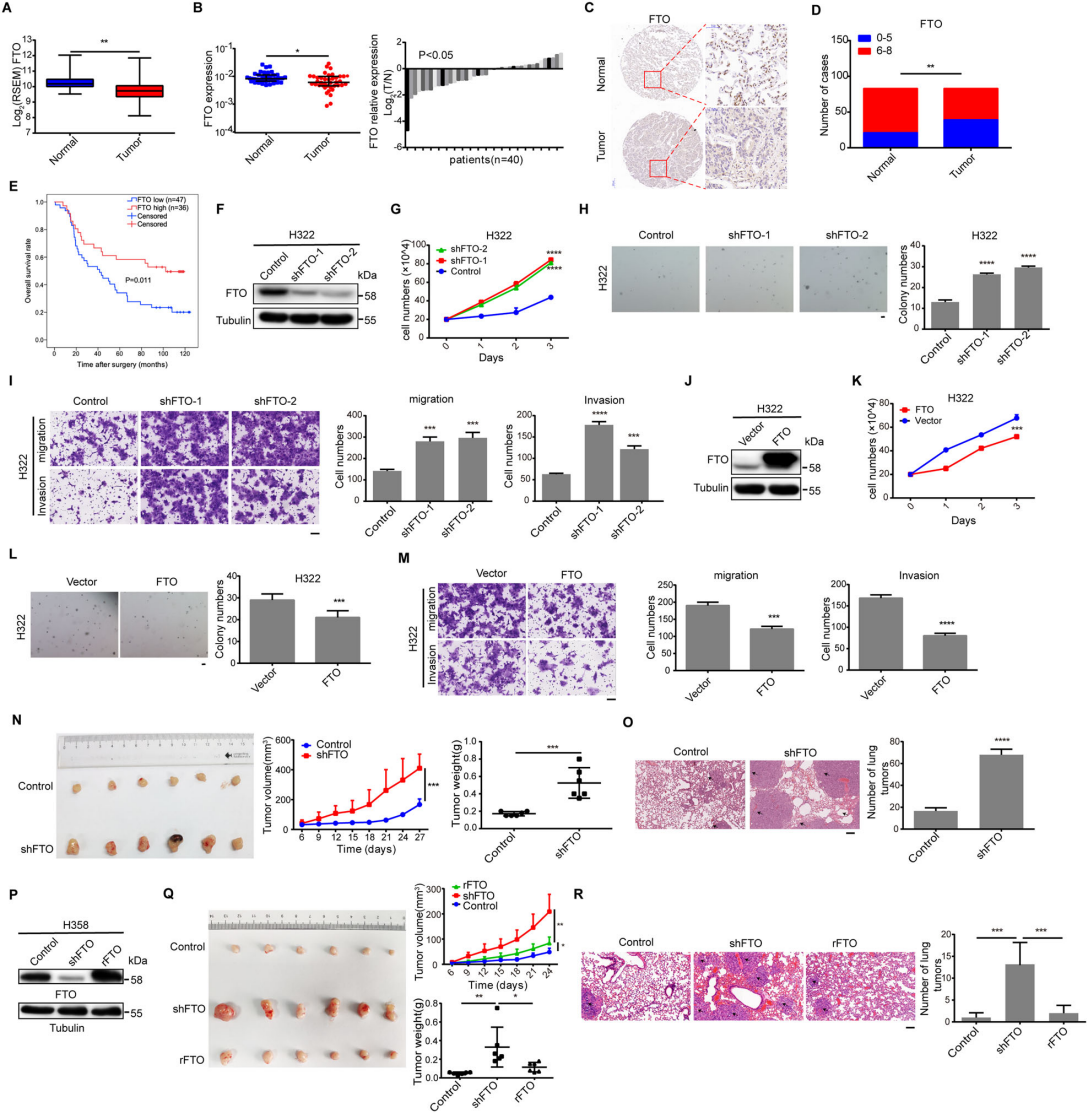
Downregulated expression of FTO is correlated with lung adenocarcinoma patients' poor survival and promotes tumor growth and metastasis. **A** The relative mRNA expression levels of *FTO* were analyzed in lung adenocarcinoma (*n* = 513) and adjacent normal tissues (*n* = 58) in the TCGA cohort. **P < 0.01. **B** Relative mRNA expression levels of *FTO* in 40 paired lung adenocarcinoma and adjacent normal tissues were determined by qPCR. T/N indicates the fold changes of *FTO* mRNA levels in tumor tissues compared to normal tissues. Bar values of <0 indicate that *FTO* mRNA is decreased in the tumor tissues. Data represent the means ± SD of the 40 paired tissues. *P < 0.05. **C** Representative IHC images for FTO protein in a paired lung adenocarcinoma and adjacent normal tissues from a tissue array were shown. Magnification, ×5 and ×30. **D** Relative FTO expression levels in 83 paired lung adenocarcinoma and adjacent normal tissues were determined by IHC scores. Pearson’s chi-square test was used (two-tailed). **E** IHC scores were used to divide the lung adenocarcinoma patients into two groups with high (score, 7 to 8) and low (score, 0 to 6) levels of FTO expression. Kaplan-Meir survival curves were compared using the log-rank test. “+” represents censored data from the patients who were alive at the last clinical follow-up. **F** H322 cells were stably transfected with a vector expressing two different FTO shRNAs. Immunoblotting analyses with the indicated antibodies were performed. **G** H322 cells with or without FTO depletion were cultured for the indicated periods of time and were harvested for cell counting. The data represent the means ± SD of triplicate samples. ****P < 0.0001. **H** H322 cells (1 × 10^4^) with or without FTO depletion by two different shRNAs (shFTO-1 and shFTO-2) were cultured in soft agar for 3 weeks. Cell clones were stained and counted from six different fields under a microscope. Scale bar: 100 μm. The data represent the means ± SD of triplicate samples. ****P < 0.0001. **I** Migration and invasion of H322 cells with or without FTO depletion was examined. Representative images of cell migration (upper left) and invasion (bottom left) are shown. The number of migrated or invaded cells was counted from 3 different fields under a microscope (right). The data represent the means ± SD. ***P < 0.001, and ****P < 0.0001. Scale bar: 100 μm. **J** H322 cells were stably transfected with a vector expressing FTO. Immunoblotting analyses with the indicated antibodies were performed. **K** H322 cells with or without FTO overexpression were cultured for the indicated periods of time and were harvested for cell counting. The data represent the means ± SD of triplicate samples. ***P < 0.001. **L** H322 cells (1 × 10^4^) with or without FTO overexpression were cultured in soft agar for 3 weeks. Cell clones were stained and counted from six different fields under a microscope. Scale bar: 100 μm. The data represent the means ± SD of triplicate samples. ***P < 0.001. **M** Migration and invasion of H322 cells with or without FTO overexpression were examined. Representative images of cell migration (upper left) and invasion (bottom left) are shown. The number of migrated or invaded cells was counted from 3 different fields under a microscope (right). The data represent the means ± SD. ***P < 0.001, and ****P < 0.0001. Scale bar: 100 μm. **N** H358 cells with or without FTO depletion were subcutaneously implanted into the flank regions of nude mice (*n* = 6). Four weeks later, tumor sizes (left), volumes (middle), and weights (right) were measured or calculated. Data represent the means ± SD of 6 mice in each group. *P < 0.05, **P < 0.01, and ***P < 0.001. **O** H358 cells with or without FTO depletion were injected into the tail veins of NOD/SCID mice (*n* = 6). The mouse lungs were stained by H&E. Representative images of metastatic tumors in the lungs are shown. The black arrows indicate metastatic tumors. Scale bar: 100 μm. The number of tumors in the lung was counted. Data represent the means ± SD of 6 mice in each group. ****P < 0.0001. **P** H358 cells with or without FTO depletion or with a reconstituted expression of an RNAi-resistant FTO (rFTO) were analyzed by immunoblotting assay with the indicated antibodies. **Q** H358 cells with or without FTO depletion or with a reconstituted expression of an RNAi-resistant FTO (rFTO) were subcutaneously implanted into the flank regions of nude mice (*n* = 6). Three weeks later, tumor sizes (left), volumes (up), and weights (down) were measured or calculated. Data represent the means ± SD of 6 mice in each group. *P < 0.05, **P < 0.01. **R** H358 cells with or without FTO depletion or with a reconstituted expression of an RNAi-resistant FTO (rFTO) were injected into the tail veins of NOD/SCID mice (*n* = 6). The mouse lungs were stained by H&E. Representative images of metastatic tumors in the lungs are shown. The black arrows indicate metastatic tumors. Scale bar: 100 μm. The number of tumors in the lung was counted. Data represent the means ± SD of 6 mice in each group. ***P < 0.001.

Lung cancer, among which non-small-cell lung carcinoma (NSCLC) accounts for 85% of cases, is the most commonly occurring cancer and the leading cause of cancer death^34,35^. As a major type of non-small-cell lung carcinoma, lung adenocarcinoma (LUAD) remains one of the most aggressive and fatal tumor types^36^. To determine the expression of FTO in lung adenocarcinoma, we analyzed TCGA data and revealed that FTO expression levels were much lower in lung adenocarcinoma tissues than in their adjacent normal tissues (Fig. 1A). To validate this finding, we examined 40 pairs of lung adenocarcinoma tissues and their adjacent normal tissues by real-time quantitative reverse transcription-polymerase chain reaction (qRT-PCR) and obtained similar findings (Fig. 1B). Consistently, immunohistochemistry (IHC) of 83 paired tissue arrays showed that nuclear FTO expression levels were lower in the lung adenocarcinoma tissues than in their adjacent normal tissues (Fig.1C, D). Kaplan-Meier analysis showed that the patients with low FTO expression had poor overall survival (Fig. 1E). These results indicate that the FTO expression is downregulated in lung adenocarcinoma and correlated with poor survival of the patients with this disease.

To determine the role of FTO in lung adenocarcinoma progression, we depleted FTO expression in H322 (Fig. 1F) and H358 (Fig. S1A) lung adenocarcinoma cells with two different shRNAs (shFTO-1 and shFTO-2). We showed that FTO depletion significantly enhanced proliferation (Figs. 1G, S1B) and anchorage-independent growth (Figs. 1H, S1C) of these cells in soft agar. In addition, we synchronized the cells at the G2/M phase by thymidine and nocodazole treatment and released them to enter the cell cycle (Fig. S1D). We showed that FTO depletion promoted the cell cycle progression, reflected by the increased number of cells with incorporated BrdU (Fig. S1E). In addition, FTO depletion accelerated the migration and invasion of H322 (Fig. 1I) and H358 (Fig. S1F) cells. In contrast, overexpression of FTO (Figs. 1J, S1G) significantly inhibited proliferation (Figs. 1K, S1H), anchorage-independent growth (Figs. 1L, S1I), migration, and invasion of H322 (Fig. 1M) and H358 (Fig. S1J) cells.

We next subcutaneously injected H358 cells with or without FTO depletion into athymic nude mice. As shown in Fig. 1N, FTO depletion significantly promoted tumor growth. Injection of these cells into the tail veins of mice showed that FTO depletion enhanced lung metastasis (Fig. 1O). Notably, the enhanced tumor growth and metastasis were abrogated by reconstituted expression of an RNAi-resistant (r) Flag-tagged FTO (rFTO) in H358 cells. (Fig. 1P–R). These results indicate that FTO downregulation in lung adenocarcinoma cells promotes tumor growth and metastasis.

### Wnt signaling induces the binding of EZH2 to β-catenin, leading to increased H3K27me3 in the *FTO* promoter region and the subsequent inhibition of FTO expression

**Fig. 2:**
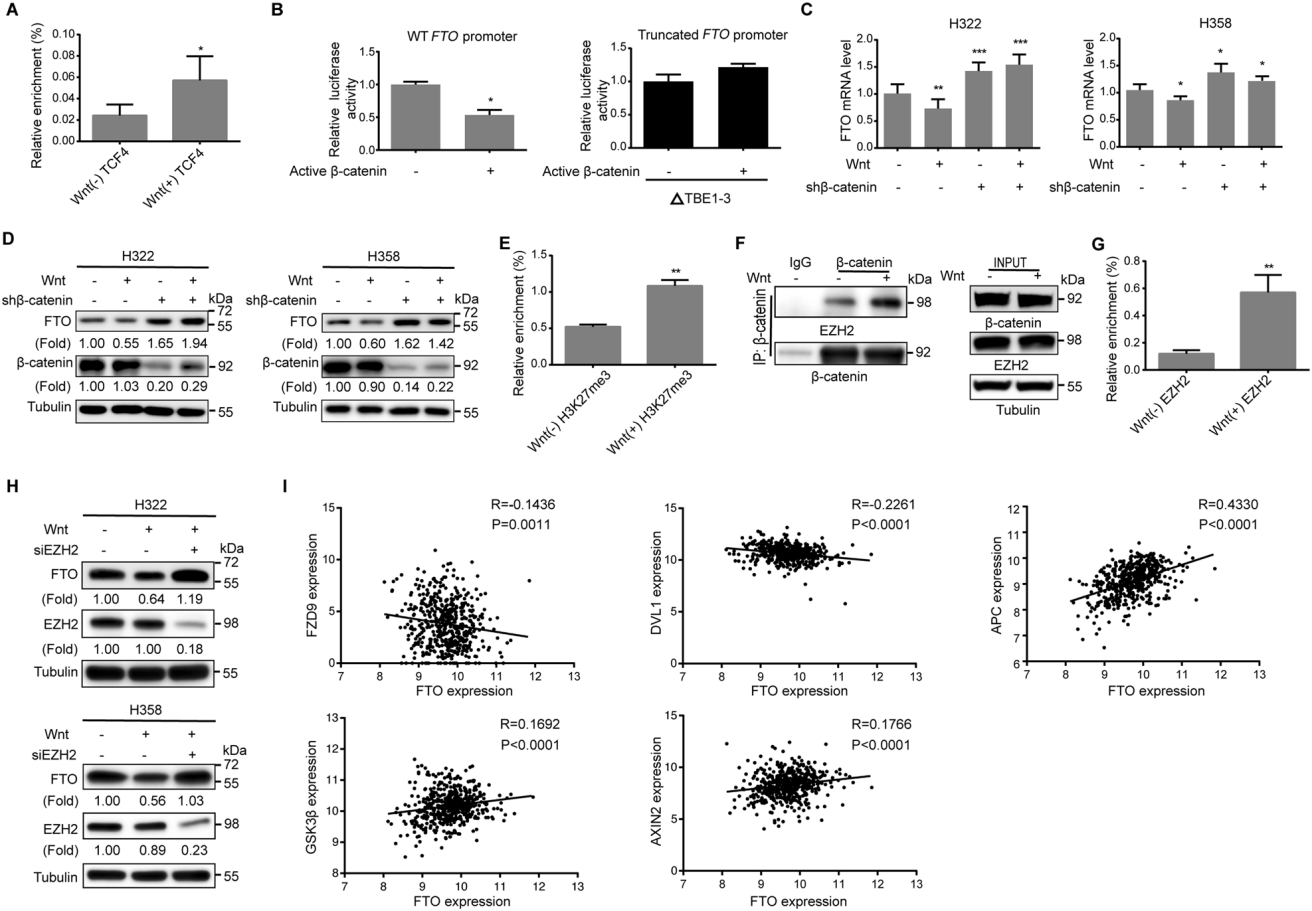
Wnt signaling induces the binding of EZH2 to β-catenin, leading to increased H3K27me3 in the *FTO* promoter region and subsequent inhibition of FTO expression. **A** H322 cells were treated with or without Wnt-3a for 8 h. ChIP analyses using an anti-TCF4 antibody and qPCR with the primer against the FTO promoter regions were performed. Data represent the means ± SD of triplicate samples. *P < 0.05. **B** A luciferase reporter vector with a WT or truncated *FTO* promoter was stably expressed in 293T cells. These cells were transfected with a control vector or a vector expressing active β-catenin. Relative luciferase activities were determined. ΔTBE1–3: deletion of the three TBEs in the *FTO* promoter. Data represent the means ± SD of triplicate samples. *P < 0.05. **C**, **D** H322 and H358 cells with or without β-catenin depletion were treated with or without Wnt-3a for 8 h. *FTO* mRNA (**C**) and protein (**D**) expression was determined by qPCR and immunoblotting analyses with the indicated antibodies, respectively. Data represent the means ± SD of triplicate samples. *P < 0.05, **P < 0.01, and ***P < 0.001. **E** H322 cells were treated with or without Wnt-3a for 8 h, ChIP analyses using an anti-H3K27me3 antibody and qPCR with the primer against the *FTO* promoter regions were performed. Data represent the means ± SD of triplicate samples. **P < 0.01. **F** H322 cells were treated with or without Wnt-3a for 8 h. Immunoprecipitation and immunoblotting analyses were performed with the indicated antibodies. **G** H322 cells were treated with or without Wnt-3a for 8 h. ChIP analyses using an anti-EZH2 antibody and qPCR with the primer against the *FTO* promoter regions were performed. Data represent the means ± SD of triplicate samples. **P < 0.01. **H** H322 and H358 cells with or without EZH2 depletion were stimulated with or without Wnt-3a for 8 h. Immunoblotting analyses were performed with the indicated antibodies. **I** The correlation of the mRNA levels of *FTO* with the mRNA levels of *FZD9*, *DVL1*, *APC*, *GSK3β*, and *AXIN2* was analyzed in lung adenocarcinoma (*n* = 513) from the TCGA cohort. Pearson’s correlation test was used (two-tailed).

To determine the mechanism underlying the downregulation of FTO in lung adenocarcinoma cells, we analyzed the *FTO* promoter sequence with PROMO software and identified three potential LEF/TCF-binding elements (TBE) (Fig. S2A) that are closely located and can be recognized by the β-catenin/LEF/TCF complex in response to Wnt signaling^37–40^. Chromatin immunoprecipitation (ChIP) assays demonstrated that Wnt stimulation increased the binding of TCF4 to the promoter region of *FTO* (Fig. 2A). Luciferase reporter analyses showed that luciferase activity driven by the wild-type (WT) *FTO* promoter was suppressed by the expression of constitutively active β-catenin^41^ (Fig. 2B, left panel). However, this suppression was abrogated by the deletion of these three TBEs in the *FTO* promoter (Fig. 2B, right panel), suggesting that activation of β-catenin/LEF/TCF signaling suppressed *FTO* promoter activity. Consistent results showed that Wnt treatment of H322 and H358 lung adenocarcinoma cells reduced the mRNA (Fig. 2C) and protein (Fig. 2D) levels of *FTO*. This reduction was abrogated by depletion of β-catenin, which enhanced FTO expression (Fig. 2C, D). These results indicate that Wnt-induced β-catenin transactivation increases the binding of the β-catenin/LEF/TCF complex to the promoter region of *FTO* and reduces FTO expression.

Histone methylations play an instrumental role in the regulation of gene transcription, and the trimethylation of histone H3 lysine (K) 27 (H3K27me3) and dimethylation of histone H3 K9 (H3K9me2) are marks of transcription repression^42^. ChIP analyses with anti-H3K27me3 and anti-H3K9me2 antibodies showed that Wnt stimulation significantly enriched H3K27me3 (Fig. 2E) but not anti-H3K9me2 (Fig. S2B) in the TBE regions of the *FTO* promoter. EZH2 histone methyltransferase is known to catalyze H3K27me3^43^. Wnt treatment enhanced the binding of EZH2 to β-catenin (Fig. 2F) and the TBE regions of the *FTO* promoter in response to Wnt stimulation (Fig. 2G), as detected by coimmunoprecipitation and ChIP assays, respectively. Depletion of EZH2 blocked Wnt-suppressed FTO expression (Fig. 2H). These results indicate that Wnt signaling induces the binding of EZH2 to β-catenin, leading to increased H3K27me3 in the promoter region of *FTO* and to subsequent inhibition of FTO expression.

We next examined the clinical relevance of Wnt signaling component expression and FTO expression by TCGA analysis. We found that expression of a positive regulator of Wnt signaling Frizzled (FZD)9 and DVL1 was negatively correlated with FTO expression whereas expression of negative regulators of Wnt signaling APC, GSK3β, and AXIN2 was positively correlated with FTO expression in lung adenocarcinoma (Fig. 2I). These results further supported that active Wnt signaling induces downregulation of FTO expression in lung adenocarcinoma.

### FTO downregulation increases m^6^A modifications of *MYC* mRNA, thereby enhancing c-Myc expression

**Fig. 3:**
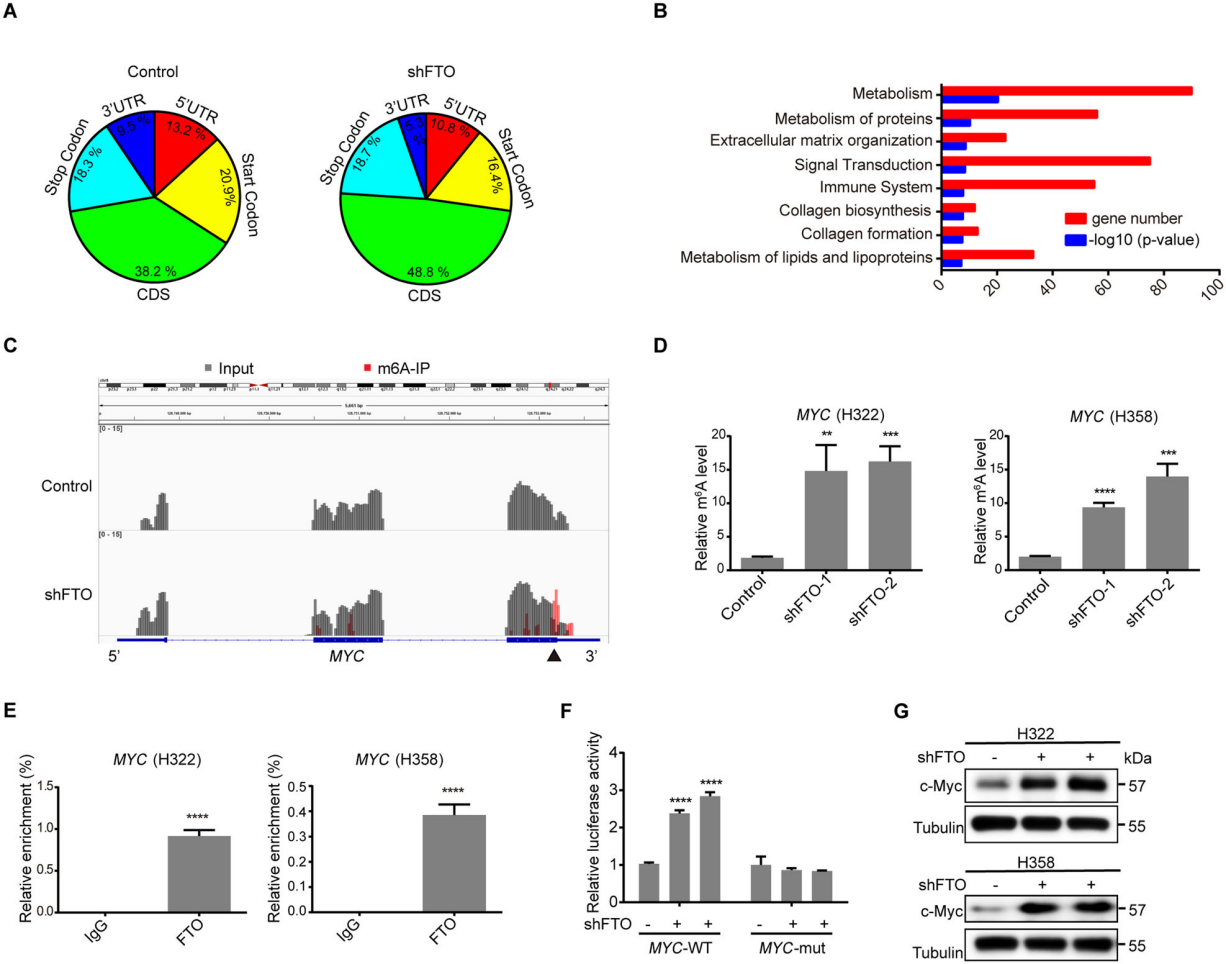
FTO downregulation increases m^6^A of *MYC* mRNA, thereby enhancing c-Myc expression. **A** m^6^A RNA sequencing (m^6^A-seq) of H322 cells with or without FTO depletion was conducted. The proportions of m^6^A peak distribution in the 5′ untranslated region (5′UTR), start codon, coding region (CDS), stop codon, and the 3′ untranslated region (3′UTR) across the entire set of mRNA transcripts were calculated. **B** m^6^A RNA sequencing (m^6^A-seq) of H322 cells with or without FTO depletion was conducted. Reactome pathway analysis of genes with increased m^6^A abundance (fold-change>4) in H322 cells with FTO depletion was performed. **C** m^6^A RNA sequencing (m^6^A-seq) of H322 cells with or without FTO depletion was conducted. The m^6^A enrichment on *MYC* mRNA is shown. A solid triangle indicates the region for qPCR analyses. **D** Methylated RNA in H322 (left) and H358 (right) cells with or without FTO depletion was immunoprecipitated with an m^6^A antibody followed by qPCR analyses with primers against *MYC* mRNA. Data represent the means ± SD of triplicate samples. **P < 0.01, ***P < 0.001, and ****P < 0.0001. **E** RIP analyses of H322 and H358 cells were performed with an anti-FTO antibody followed by qPCR analyses with primers against *MYC* mRNA. Data represent the means ± SD of triplicate samples. ****P < 0.0001. **F** Luciferase vectors with WT or mutated m^6^A nucleotides in the *MYC* gene were transfected into 293T cells with or without FTO depletion. Luciferase activity was measured. Data represent the means ± SD of triplicate samples. ****P < 0.0001. **G** H322 and H358 cells with or without FTO depletion were analyzed by immunoblotting analyses with the indicated antibodies.

To determine the regulation of the epitranscriptome by FTO in lung adenocarcinoma, we performed m^6^A RNA sequencing (m^6^A-seq) on H322 cells with or without FTO depletion. We showed that m^6^A peaks primarily occurred in a sequence context as GGAC (*P* = 3.5e10–41) (Fig. S3A) and were mostly enriched in the coding region (CDS) of mRNAs (Fig. 3A), which are consistent with previous publications^13,33^. FTO depletion increased m^6^A abundance in the mRNAs of 556 genes and decreased m^6^A abundance in the mRNAs of 319 genes (fold-change >4, Table S4). To identify FTO-targeted genes, we performed reactome pathway analysis. We found that m^6^A was substantially increased in genes involved in metabolic pathways (Fig. 3B), which included *MYC*, a transcription factor critical for metabolic gene expression^18,44,45^. m^6^A-seq showed that the m^6^A levels were significantly enhanced in the last exon near the termination codon region of *MYC* following FTO depletion (Fig. 3C). Methylated RNA immunoprecipitation with an m^6^A antibody, which was followed by qPCR analyses, showed that FTO depletion increased m^6^A levels of *MYC* mRNA (Fig. 3D), FTO overexpression decreased m^6^A levels of *MYC* mRNA in both H322 and H358 cells (Fig. S3B). Consistently, RIP analyses with an anti-FTO antibody showed that FTO bound to *MYC* mRNA in the tumor cells (Fig. 3E). These results strongly suggest that FTO binds to *MYC* mRNA and decreases the m^6^A level of *MYC* mRNA.

To determine the effect of FTO-dependent m^6^A regulation on c-Myc expression, we constructed a luciferase reporter gene with an integrated CDS sequence containing the WT or mutated m^6^A sites from the 3′ end of *MYC* mRNA (Fig. S3C). Luciferase assays showed that FTO depletion largely increased the activity of luciferase with WT, but not mutated, *MYC* (Fig. 3F). Consistent with this finding, FTO depletion, which did not affect *MYC* mRNA expression (Fig. S3D), increased c-Myc expression (Fig. 3G). On the contrary, FTO overexpression, which had no effect on *MYC* mRNA expression (Fig. S3E), decreased c-Myc expression (Fig. S3F). These results indicate that FTO downregulation increases the m^6^A level of *MYC* mRNA, thereby enhancing c-Myc expression.

### YTHDF1 binds to m^6^A-modified *MYC* mRNA and promotes its translation

**Fig. 4:**
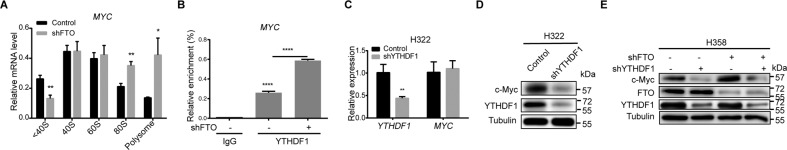
YTHDF1 binds to m^6^A-modified *MYC* mRNA and promotes its translation. **A** *MYC* mRNA level in different pools of ribosomes from H322 cells with or without FTO depletion was determined by polysome profiling and qPCR assays. Data represent the means ± SD of triplicate samples. *P < 0.05, **P < 0.01. **B** RIP analyses of H322 cells were performed with an anti-YTHDF1 antibody followed by qPCR analyses with primers against *MYC* mRNA. Data represent the means ± SD of triplicate samples. ****P < 0.0001. **C** qPCR analysis was performed for mRNA levels of *YTHDF1* and *MYC* in H322 cells with or without YTHDF1 depletion. Data represent the means ± SD of triplicate samples. **P < 0.01. **D** H322 cells with or without YTHDF1 depletion were analyzed by immunoblotting assays with the indicated antibodies. **E** *YTHDF1* shRNA was expressed in H358 cells with or without FTO depletion. Immunoblotting analyses were performed with the indicated antibodies.

Given that FTO did not affect *MYC* mRNA expression, we next determined whether FTO regulated c-Myc expression through regulation of *MYC* mRNA translation. Polysome profiling analyses of the association of *MYC* mRNA with ribosomes showed that *MYC* mRNA was increased in the translating pool of ribosomes (80S monosomes and polysomes), but not in subunits smaller than 40S, 40S, and 60S (Fig. 4A). These results strongly suggest that FTO depletion promotes the translation of *MYC* mRNA.

It is known that the YTHDF1 protein, as an m^6^A reader, promotes the translation of m^6^A-modified mRNAs^7–9^. To determine whether YTHDF1 proteins play a role in the regulation of c-Myc expression, we performed RIP analyses with an anti-YTHDF1 antibody and showed that FTO depletion enhanced the binding of YTHDF1 to *MYC* mRNA (Fig. 4B). Although depletion of YTHDF1 did not affect *MYC* mRNA expression (Figs. 4C, S4A), it reduced c-Myc expression in both H322 and H358 cells (Figs. 4D, S4B). Notably, FTO depletion-induced upregulation of c-Myc was abrogated by YTHDF1 depletion (Figs. 4E, S4C). These results strongly suggest that YTHDF1 binds to *MYC* mRNA with high m^6^A levels that are induced by FTO downregulation, and that binding promotes *MYC* mRNA translation.

### FTO downregulation-induced c-Myc expression promotes tumor cell glycolysis, growth, migration, invasion, and tumorigenesis in mice

**Fig. 5:**
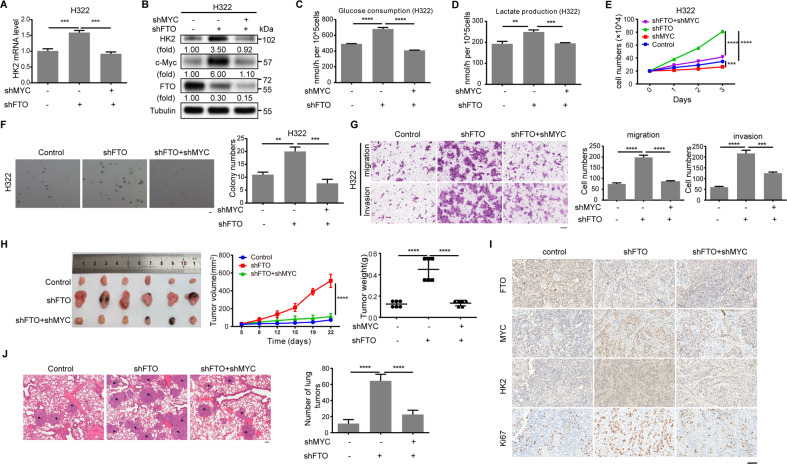
FTO downregulation-induced c-Myc expression promotes tumor cell glycolysis, growth, invasion, and tumorigenesis in mice. **A** qPCR analysis of H322 cells with or without *FTO* shRNA expression or combined expression of *FTO* shRNA and *MYC* shRNA was performed. Data represent the means ± SD of triplicate samples. ***P < 0.001. **B** Immunoblotting analysis of H322 cells with or without *FTO* shRNA expression or combined expression of *FTO* shRNA and *MYC* shRNA was performed with the indicated antibodies. **C**, **D** Glucose consumption (**C**) and lactate production (**D**) of H322 cells with or without expression of *FTO* shRNA or combined expression of *FTO* shRNA and *MYC* shRNA were determined. Data represent the means ± SD of triplicate samples. **P < 0.01, ***P < 0.001, ****P < 0.0001. **E** H322 cells with or without *FTO* shRNA or *MYC* shRNA were cultured for the indicated periods of time and were harvested for cell counting. The data represent the means ± SD of triplicate samples. ****P < 0.0001. **F**, **G** The anchorage-independent growth of H322 cells with or without expression of FTO shRNA or combined expression of *FTO* shRNA and *MYC* shRNA was measured by soft agar assays (**F**). Scale bar: 100 μm. The data represent the means ± SD of triplicate samples. The migration and invasion of the indicated cells were measured (**G**). Scale bar: 100 μm. The number of migrated and invaded cells were counted from 3 different fields under a microscope. The data represent the means ± SD. **P < 0.01, ***P < 0.001, and ****P < 0.0001. **H**, **I** H358 cells with or without expression of *FTO* shRNA or combined expression of *FTO* shRNA and *MYC* shRNA were subcutaneously implanted into the flank regions of nude mice (*n* = 6). Photographs of tumors, tumor growth curves and tumor weights are shown or calculated (**H**). Data represent the means ± SD of 6 mice in each group. IHC staining of tumor tissues was performed with indicated antibodies. Representative images are shown (**I**). Scale bar: 100 μm. **P < 0.01, ***P < 0.001, and ****P < 0.0001. **J** H358 cells with or without expression of *FTO* shRNA or combined expression of *FTO* shRNA and *MYC* shRNA were injected into the tail veins of NOD/SCID mice (*n* = 5). Mouse lungs were stained by H&E. Representative images of metastatic tumors in the lungs are shown. The black arrows indicate metastatic tumors. Scale bar: 100 μm. The number of tumors in the lung was counted. Data represent the means ± SD of 5 mice in each group. ****P < 0.0001.

c-Myc plays an important role in cancer metabolism, including aerobic glycolysis^46,47^. mRNA sequencing of H322 cells with or without FTO depletion showed that FTO depletion largely increased the mRNA level of glycolytic gene hexokinase 2 (HK2) (Table S5). As expected, FTO depletion increased the mRNA and protein levels of HK2 in H322 and H358 cells (Fig. S5A, B). Notably, these increases were abrogated by c-Myc depletion (Fig. 5A, B, Fig. S5C, D). Correspondingly, FTO depletion resulted in increased glucose uptake (Figs. 5C, S5E), lactate production (Figs. 5D, S5F), cell proliferation (Figs. 5E, S5G, H), cell growth in soft agar (Figs. 5F, S5I), migration, and invasion (Figs. 5G, S5J) of the tumor cells; these increases were all inhibited by c-Myc depletion. These results indicate that FTO downregulation-induced c-Myc expression promotes tumor cell glycolysis, growth, migration, and invasion.

We next subcutaneously injected H358 cells with or without FTO depletion or combined FTO and c-Myc depletion into athymic nude mice. We showed that FTO depletion-promoted tumor growth was blunted by c-Myc depletion (Fig. 5H). Immunohistochemistry (IHC) staining of the tumor tissues showed that FTO depletion increased expression of c-Myc, HK2. and proliferation marker protein Ki67^48^. Of note, the FTO depletion-enhanced these protein expressions were largely reduced by c-Myc depletion (Fig. 5I). In addition, FTO expression levels were inversely correlated with Wnt-3a levels in tumor tissues (Fig. S5K), further supporting the role of the activated Wnt/β-catenin signaling in the repression of FTO. As expected, FTO depletion-enhanced tumor metastasis in the lung was also inhibited by c-Myc depletion (Fig. 5J). These results indicate that FTO downregulation-enhanced c-Myc expression promotes lung tumor growth and metastasis in mice.

## Discussion

**Fig. 6:**
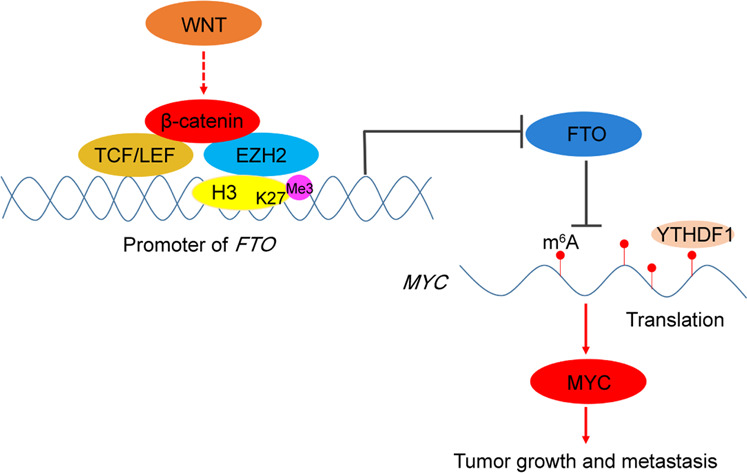
WNT/β-catenin-suppressed FTO increases c-Myc expression to promote tumor growth. A mechanism of lung adenocarcinoma growth and metastasis promoted by Wnt/β-catenin-suppressed FTO expression and a subsequently increased m^6^A of *MYC* mRNA and protein expression of c-Myc.

In addition to epigenetic regulation of gene expression, regulation of the epitranscriptome exerts another critical layer to regulate protein expression. FTO, a key demethylase that removes the m^6^A modification from mRNAs, plays instrumental roles in the regulation of many cellular functions^3–7^. However, how FTO is transcriptionally regulated, especially by oncogenic signaling, remains to be explored. We demonstrated here that FTO expression was downregulated in lung adenocarcinoma and positively correlated with overall survival. Depletion of FTO enhanced tumor cell proliferation, anchorage-independent growth, migration, invasion, and tumor formation in mice. Of special interest, we found that Wnt signaling, which is vital in lung adenocarcinoma tumorigenesis^49–51^, induced the binding of β-catenin/TCF/LEF to the TBEs of the *FTO* promoter region and suppressed FTO expression. Mechanistically, we showed that WNT signaling induced the binding of EZH2 to β-catenin, leading to an EZH2-dependent H3K27me3 increase at the *FTO* promoter region for inhibition of FTO expression. Downregulation of FTO expression enhanced m^6^A levels on mRNAs in a number of signaling pathways, including metabolic pathways, in which *MYC* is a master regulator of gene expression for glycolysis^18,44,45^. Enhanced m^6^A levels on *MYC* mRNA recruited the binding of YTHDF1 and enhanced *MYC* mRNA translation. FTO depletion and YTHDF1-dependent upregulation of c-Myc promoted tumor cell glycolysis, growth, migration, and invasion and accelerated tumor growth in mice and tumor metastasis to the lung (Fig. 6).

*MYC* gene transcription can be upregulated directly by β-catenin/TCF/LEF^18,38,45^. FTO downregulation can upregulate Wnt signaling by increasing m^6^A modification in *FZD10* mRNA^52^. In addition, m^6^A-dependent and YTHDF2-dependent decreases in *MYC* transcript stability in leukemia cells were reported^14^. We showed that Wnt stimulation suppressed FTO expression, therefore inducing m^6^A-dependent and YTHDF1-dependent increases in *MYC* mRNA translation. Given that FTO globally regulates the expression of many genes, which can be signaling context-dependent and cancer type-dependent^16^, the differential and distinct regulation of m^6^A in mRNAs downstream of the FTO exhibit wide variation and may elicit distinct cellular activities. Our finding that FTO downregulation induced by Wnt/β-catenin signaling enhanced c-Myc expression through upregulation of m^6^A of *MYC* mRNA and subsequent YTHDF1 binding revealed a novel mechanism by which lung adenocarcinoma promotes glycolysis and growth. The discovery of the novel regulation of c-Myc expression may lead to an alternative approach for the therapeutic treatment of lung adenocarcinoma.

## Supplementary information

Supplementary figure legends

Supplementary table 1

Supplementary table 2

Supplementary table 3

Supplementary table 4

Supplementary table 5

Supplementary figure 1

Supplementary figure 2

Supplementary figure 3

Supplementary figure 4

Supplementary figure 5

## Data Availability

The m^6^A-seq data has been deposited into the Gene Expression Omnibus repository under accession number GSE171472. All the other data used and/or analyzed during this study are available from the corresponding authors on reasonable request.
