# Supplementary material for: WNT/β-catenin-suppressed FTO expression increases m6A of c-Myc mRNA to promote tumor cell glycolysis and tumorigenesis
Source: Cell Death Dis. 2021 May 8;12(5):462. doi: 10.1038/s41419-021-03739-z (PMC8106678; doi:10.1038/s41419-021-03739-z)
Supplement: Supplementary file 1 — Supplementary figure legends [file 41419_2021_3739_MOESM1_ESM.docx]

**Supplementary figure legends**

**Fig. S1. FTO supresses lung adenocarcinoma cells growth and metastasis**

**(A)** H358 cells were stably transfected with a control vector or two different FTO shRNAs. Immunoblotting analyses with the indicated antibodies were performed.

**(B)** H358 cells with or without FTO depletion were cultured for the indicated periods of time and were harvested for cell counting. The data represent the means ± SD of triplicate samples. **** P<0.0001.

**(C)** H358 cells (1 × 10^4^) with or without FTO depletion by two different shRNAs (shFTO-1 and shFTO-2) were cultured in soft agar for 3 weeks. Cell clones were stained and counted from six different fields under a microscope. Scale bar: 100 μm. The data represent the means ± SD of triplicate samples. ** P<0.01, *** P<0.001.

**(D, E)** H358 cells with or without FTO depletion were arrested at G2/M phase **(D)**, and the proportion of cells with incorporated BrdU was evaluated by flow cytometry after releasing from G2/M phase synchronization **(E)**.

**(F)** Migration and invasion of H358 cells with or without FTO depletion were examined. Representative images (left) are shown. The number of migrated or invaded cells was counted from 3 different fields under a microscope (right). The data represent the means ± SD. ** P<0.01, *** P<0.001, and **** P<0.0001. Scale bar: 100 μm.

**(G)** H358 cells with ectopic FTO expression were analysed by immunoblotting assay with the indicated antibodies.

**(H)** H358 cells with or without FTO overexpression were cultured for the indicated periods of time and were harvested for cell counting. The data represent the means ± SD of triplicate samples. * P<0.05.

**(I)** H358 cells (1 × 10^4^) with or without FTO overexpression were cultured in soft agar for 3 weeks. Cell colonies were stained and counted from six different fields under a microscope. Scale bar: 100 μm. The data represent the means ± SD of triplicate samples. *** P<0.001.

**(J)** Migration and invasion of H358 cells with or without FTO overexpression were examined. Representative images (left) are shown. The number of migrated or invaded cells was counted from 3 different fields under a microscope (right). The data represent the means ± SD. *** P<0.001, and **** P<0.0001. Scale bar: 100 μm.

**Fig. S2. Wnt stimulation does not alter H3K9me2 levels**

**(A)** Three potential LEF/TCF-binding elements (TBE) on the *FTO* promoter were identified using PROMO software. The sequences and locations of these three TBEs are shown. TSS, the transcriptional start site.

**(B)** H322 cells were treated with or without Wnt-3a for 8 h. ChIP analyses using an anti-H3K9me2 antibody and qPCR with the primer against the *FTO* promoter regions were performed. Data represent the means ± SD of triplicate samples.

**Fig. S3. FTO downregulation increases m^6^A modifications of *MYC* mRNA, thereby enhancing c-Myc expression**

**(A)** The most frequent m^6^A motif detected by DREME in m^6^A-seq of H322 cells is shown. P=3.5e10-41.

**(B)** Methylated RNA in H322 (left) and H358 (right) cells with or without FTO overexpression was immunoprecipitated with an anti-m^6^A antibody followed by qPCR analyses with primers against *MYC* mRNA. Data represent the means ± SD of triplicate samples. * P<0.05, and **** P<0.0001.

**(C)** *MYC* CDS containing WT m^6^A motifs or mutated m^6^A motifs (A-T) were inserted into the Xhol site right before the stop codon of the luciferase gene.

**(D)** qPCR analysis was performed for *MYC* in H322 and H358 cells with or without FTO depletion. Data represent the means ± SD of three independent experiments.

**(E)** qPCR analysis was performed for *MYC* mRNA in H322 and H358 cells with or without FTO overexpression. Data represent the means ± SD of three independent experiments.

**(F)** H322 and H358 cells with or without FTO overexpression were analysed by immunoblotting analyses with the indicated antibodies.

**Fig. S4. YTHDF1 binds to m^6^A-modified *MYC* mRNA and promotes its translation**

**(A)** qPCR analysis was performed for *YTHDF1* and *MYC* in H358 cells with or without YTHDF1 depletion. Data represent the means ± SD of triplicate samples. **** P<0.0001.

**(B)** H358 cells with or without YTHDF1 depletion were analysed by immunoblotting assays with the indicated antibodies.

**(C)** *YTHDF1* shRNA was expressed in H322 cells with or without FTO depletion. Immunoblotting analyses were performed with the indicated antibodies.

**Fig. S5. FTO downregulation-enhanced c-Myc expression promotes tumour cell migration**

**(A-B)** qPCR analysis **(A)** and immunoblotting analysis **(B)** of H322 and H358 cells with or without FTO depletion were performed. Data represent the means ± SD of triplicate samples. **P<0.01, *** P<0.001, ****P<0.0001.

**(C-D)** qPCR analysis **(C)** and immunoblotting analysis **(D)** of H358 cells with or without expression of *FTO* shRNA or combined expression of *FTO* shRNA and *MYC* shRNA were performed. Data represent the means ± SD of triplicate samples. * P<0.05, **** P<0.0001.

**(E, F)** Glucose consumption **(E)** and lactate production **(F)** of H358 cells with or without expression of *FTO* shRNA or combined expression of *FTO* shRNA and *MYC* shRNA were determined. Data represent the means ± SD of triplicate samples. **P<0.01, *** P<0.001.

**(G)** H358 cells with or without expression of *FTO* shRNA or *MYC* shRNA were cultured for the indicated periods of time and were harvested for cell counting. The data represent the means ± SD of triplicate samples. **** P<0.0001.

**(H)** Immunoblotting analysis of H322 and H358 cells with or without expression of *FTO* shRNA or *MYC* shRNA were performed.

**(I, J)** The anchorage-independent growth of H358 cells with or without expression of FTO shRNA or combined expression of *FTO* shRNA and *MYC* shRNA was measured by soft agar assays **(I)**. Scale bar: 100 μm. The data represent the means ± SD of triplicate samples. The migration and invasion of the indicated cells were measured **(J)**. Scale bar: 100 μm. The number of migrated and invaded cells was counted from 3 different fields under a microscope. The data represent the means ± SD. *** P<0.001, and **** P<0.0001.

**(K)** H358 cells was subcutaneously implanted into the flank regions of nude mice (n=6). IHC staining of tumour tissues was performed with indicated antibodies. Representative images are shown. Scale bar: 100 μm.
