# Supplementary material for: WNT/β-catenin-suppressed FTO expression increases m6A of c-Myc mRNA to promote tumor cell glycolysis and tumorigenesis
Source: Cell Death Dis. 2021 May 8;12(5):462. doi: 10.1038/s41419-021-03739-z (PMC8106678; doi:10.1038/s41419-021-03739-z)
Supplement: Supplementary file 2 — Supplementary table 1 [file 41419_2021_3739_MOESM2_ESM.docx]

**Table S1.**

**Clinical characteristics of patients for validation by qPCR (n=40)**

| **Characteristics** | **Number of patients (%)** |
| --- | --- |
| **Gender** |  |
| Male | 17 (42.5%) |
| Female | 23 (57.5%） |
| **Age** (years) |  |
| ≤60 | 22 (55.0%） |
| >60 | 18 (45.0%） |
| **T stage** |  |
| I | 3 (7.5%) |
| II | 26 (65.0%) |
| III | 7 (17.5%) |
| IV | 4 (10.0%） |
| **Node metastasis** |  |
| No | 20 (50.0%） |
| Yes | 20 (50.0%) |
| **Distant metastasis** |  |
| No | 40 (100.0%) |
| Yes | 0 (0.0%) |
| **TNM stage** |  |
| I | 15 (37.5%) |
| II | 5 (12.5%) |
| III | 19 (47.5%) |
| IV | 1 (2.5%) |

Abbreviations: TNM stage, tumor-node-metastasis stage.

**Clinical characteristics of patients for validation by IHC (n=83)**

| **Characteristics** | **Number of patients (%)** |
| --- | --- |
| **Gender** |  |
| Male | 55 (66.3%) |
| Female | 28 (33.7%） |
| **Age** (years) |  |
| ≤60 | 43 (51.8%） |
| >60 | 40 (48.2%） |
| **T stage** |  |
| I | 5 (6.0%) |
| II | 52 (62.6%) |
| III | 13 (15.7%) |
| IV | 13 (15.7%） |
| **Node metastasis** |  |
| No | 35 (42.2%） |
| Yes | 48 (57.8%) |
| **Distant metastasis** |  |
| No | 83 (100.0%) |
| Yes | 0 (0.0%) |
| **TNM stage** |  |
| I | 17 (20.5%) |
| II | 24 (28.9%) |
| III | 38 (45.8%) |
| IV | 4 (4.8%) |

Abbreviations: TNM stage, tumor-node-metastasis stage.
