# Supplementary material for: WNT/β-catenin-suppressed FTO expression increases m6A of c-Myc mRNA to promote tumor cell glycolysis and tumorigenesis
Source: Cell Death Dis. 2021 May 8;12(5):462. doi: 10.1038/s41419-021-03739-z (PMC8106678; doi:10.1038/s41419-021-03739-z)
Supplement: Supplementary file 3 — Supplementary table 2 [file 41419_2021_3739_MOESM3_ESM.docx]

**Table S2. Primers used in this study.**

| **Genes** | **Primers (5’ to 3’)** |
| --- | --- |
| ACTB | F: CTACCTCATGAAGATCCTCACCGA |
|  | R: TTCTCCTTAATGTCACGCACGATT |
| FTO | F: GACCTGTCCACCAGATTTTCA |
|  | R: AGCAGAGCAGCATACAACGTA |
| MYC | F: TGTCCTGAGCAATCACCTATG |
|  | R: AGTCCAATTTGAGGCAGTTTAC |
| YTHDF1 | F: ATCAAACTTCCCCTTCCACTT |
|  | R: CTACCTGCTCTTCAGCGTCAA |
| GAPDH | F: CCTGGTATGACAACGAATTTG |
|  | R: CAGTGAGGGTCTCTCTCTTCC |
| HK2 | F: TGCCACCAGACTAAACTAGACG |
|  | R: CCCGTGCCCACAATGAGAC |
