# Supplementary material for: WNT/β-catenin-suppressed FTO expression increases m6A of c-Myc mRNA to promote tumor cell glycolysis and tumorigenesis
Source: Cell Death Dis. 2021 May 8;12(5):462. doi: 10.1038/s41419-021-03739-z (PMC8106678; doi:10.1038/s41419-021-03739-z)
Supplement: Supplementary file 4 — Supplementary table 3 [file 41419_2021_3739_MOESM4_ESM.docx]

**Table S3.** **Sequence of shRNAs for knockdown used in this study.**

|  | **Sequence of shRNAs** |
| --- | --- |
| shβ-catenin | 5’-CCGGGAGTGACTCAAGAAGTGAAGACTCGAGTCTTCAC  TTCTTGAGTCACTCTTTTTTG-3’ |
| shFTO1 | 5’-CCTGAACACCAGGCTCTTTCGAAAAAGAGCCTGGTGTT  CAGG-3’ |
| shFTO2 | 5’-GCAAGAGCAGTGGAGACTTCGAAAAGTCTCCACTGCTC  TTGC-3’ |
| shYTHDF1 | 5’-GGAAACGTCCAGCCTAATTCTCGAAAGAATTAGGCTGGA  CGTTTCC-3’ |
| shMYC | 5’-GGAAACGACGAGAACAGTTGACGAATCAACTGTTCTCG  TCGTTTCC-3’ |
