# Supplementary material for: WNT/β-catenin-suppressed FTO expression increases m6A of c-Myc mRNA to promote tumor cell glycolysis and tumorigenesis
Source: Cell Death Dis. 2021 May 8;12(5):462. doi: 10.1038/s41419-021-03739-z (PMC8106678; doi:10.1038/s41419-021-03739-z)
Supplement: Supplementary file 6 — Supplementary table 5 [file 41419_2021_3739_MOESM6_ESM.docx]

**Table S5. c-Myc downstream gene expression in the presence or absence of FTO depletion in H322 cells (mRNA levels).**

| **Gene** | **Control (FPKM)** | **shFTO (FPKM)** | **Fold change (shFTO/Control)** |
| --- | --- | --- | --- |
| HK2 | 14.49 | 27.7 | 1.91 |
| ENO1 | 1570.59 | 1349.26 | 0.86 |
| LDHA | 1332.01 | 1214.51 | 0.91 |
| GLUT1 | 151.91 | 169.11 | 1.11 |
| PKM | 4531.93 | 4211.68 | 0.93 |
| MCT1 | 18.147 | 22.09 | 1.22 |
| MCT2 | 0 | 0 | - |
| GAPDH | 4886.1 | 4414.94 | 0.90 |
| TPI | 1105.93 | 1283.11 | 1.16 |
| SLC1A5 | 125.06 | 102.21 | 0.82 |
| SLC38A5 | 0.12945 | 0.028474 | 0.22 |
| ACACA | 25.52 | 28.11 | 1.1 |
| FASN | 119.10 | 120.77 | 1.01 |
| SCD | 46.87 | 51.39 | 1.1 |
