# Supplementary figures and images for: WNT/β-catenin-suppressed FTO expression increases m6A of c-Myc mRNA to promote tumor cell glycolysis and tumorigenesis
Source: Cell Death Dis. 2021 May 8;12(5):462. doi: 10.1038/s41419-021-03739-z (PMC8106678; doi:10.1038/s41419-021-03739-z)

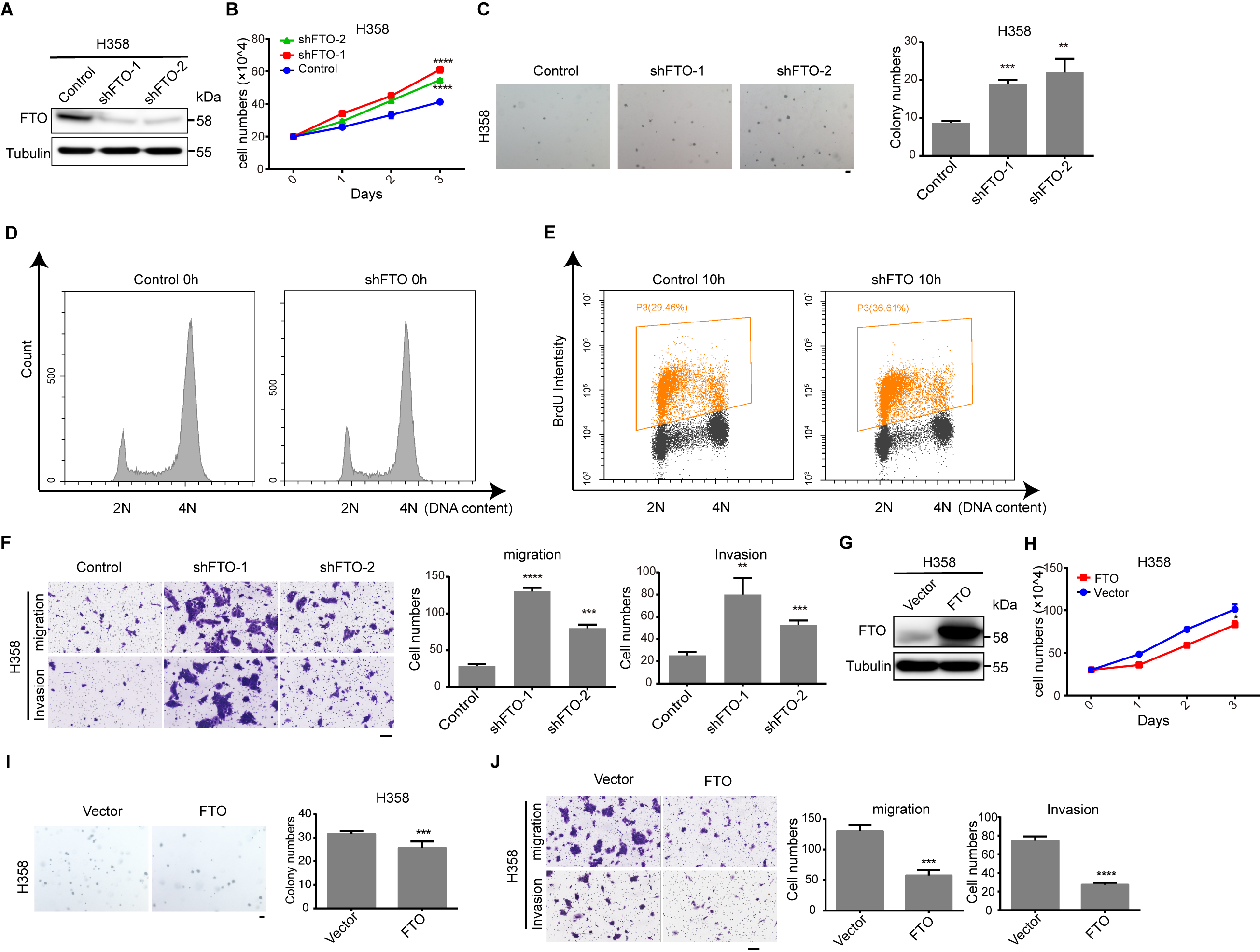

Supplement: Supplementary file 7 — Supplementary figure 1 [file 41419_2021_3739_MOESM7_ESM.tif]

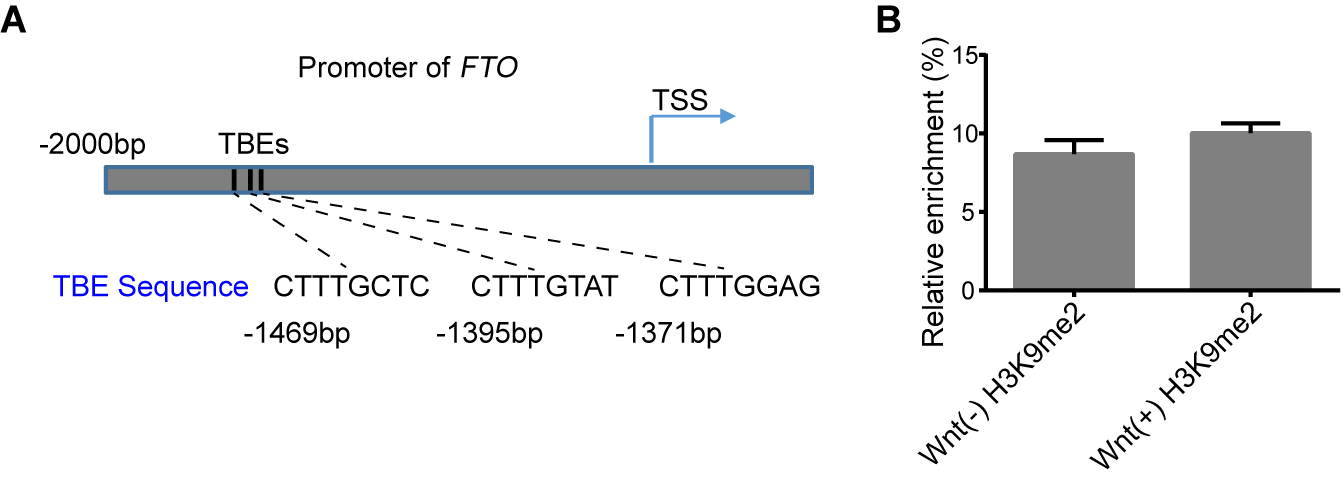

Supplement: Supplementary file 8 — Supplementary figure 2 [file 41419_2021_3739_MOESM8_ESM.tif]

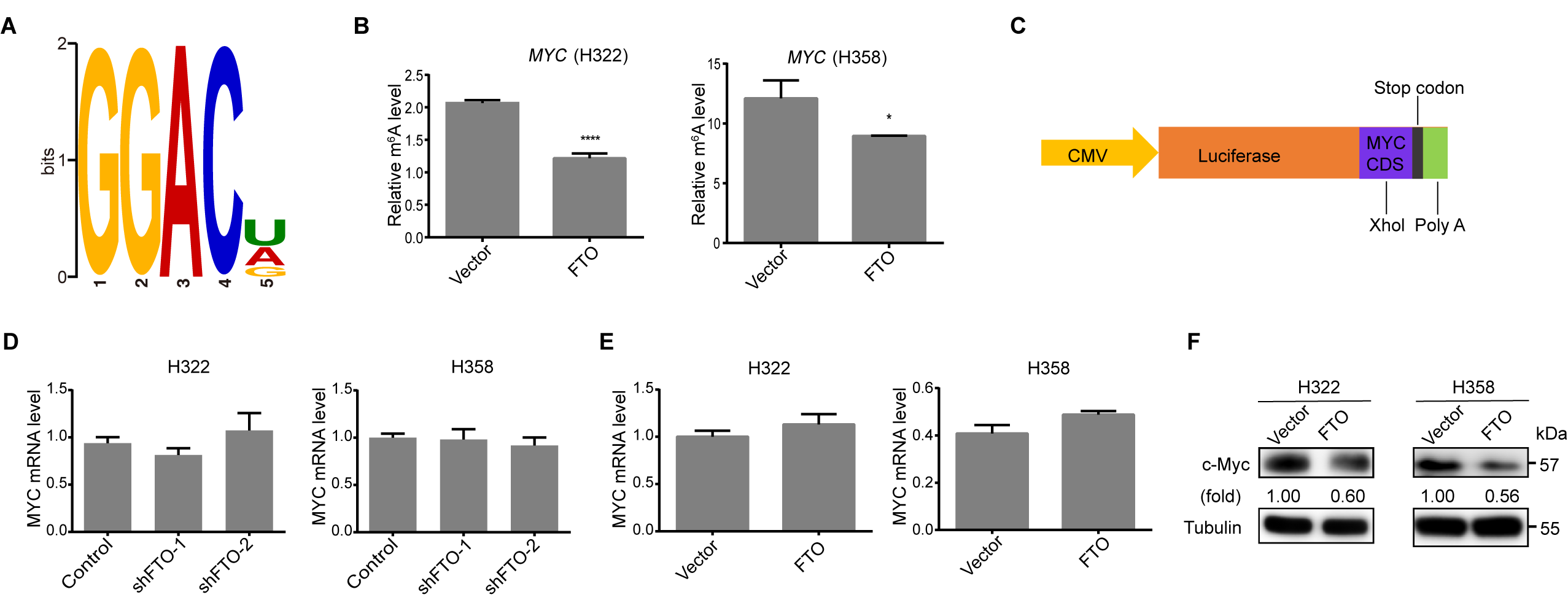

Supplement: Supplementary file 9 — Supplementary figure 3 [file 41419_2021_3739_MOESM9_ESM.tif]

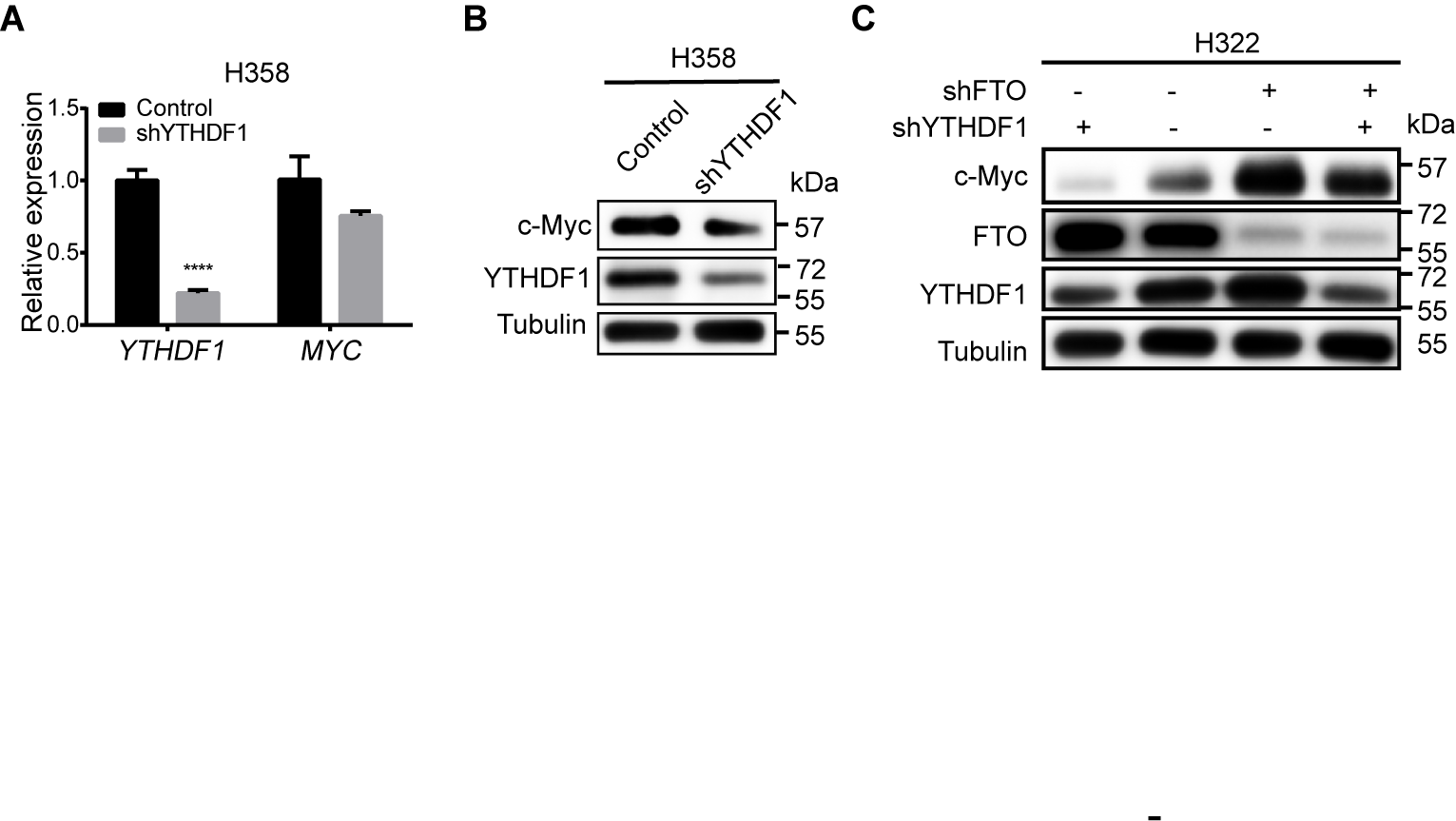

Supplement: Supplementary file 10 — Supplementary figure 4 [file 41419_2021_3739_MOESM10_ESM.tif]

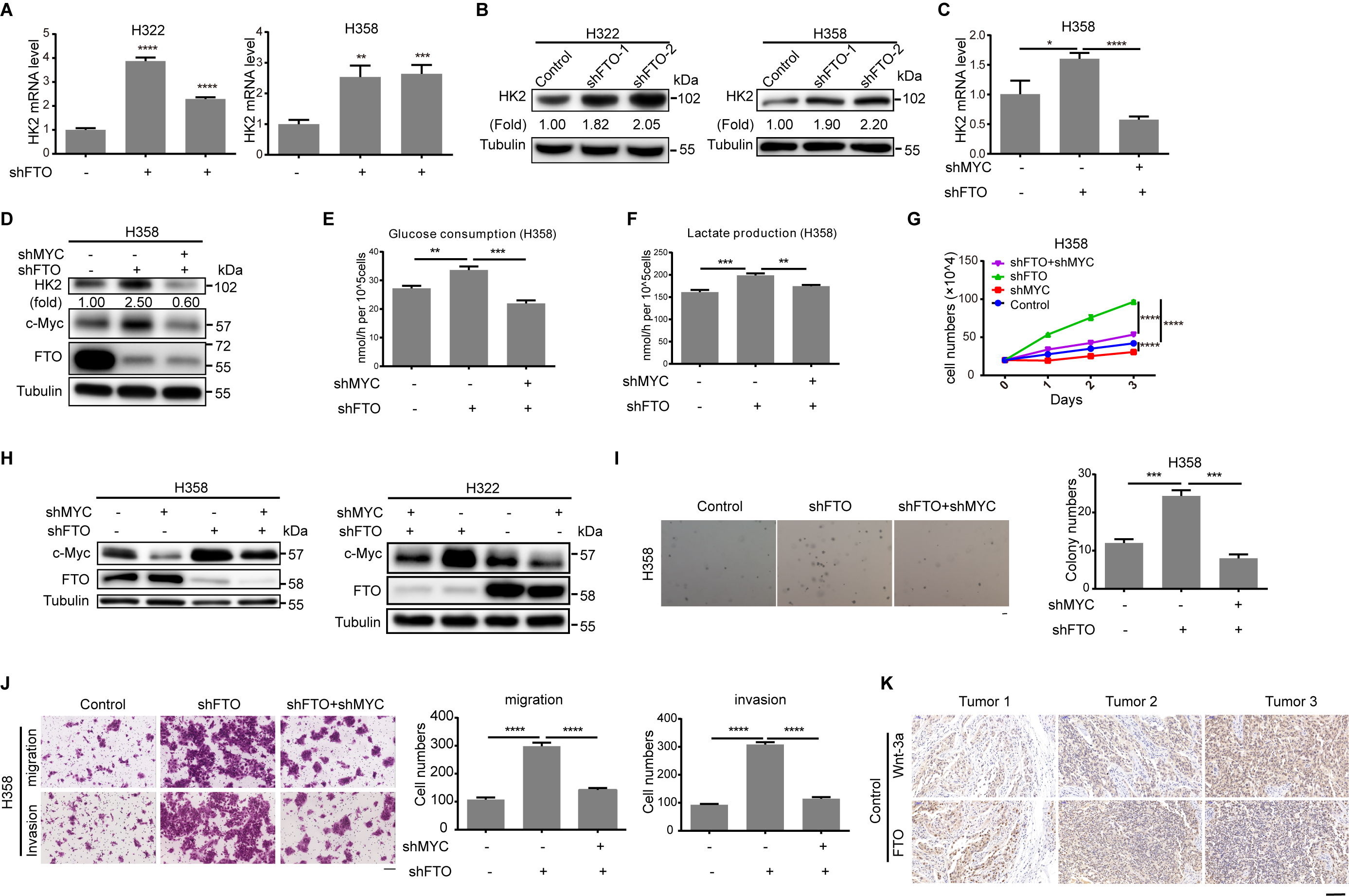

Supplement: Supplementary file 11 — Supplementary figure 5 [file 41419_2021_3739_MOESM11_ESM.tif]
